# COVID-19 Infection in a Patient With Sigmoid Diverticulitis: Viral Diverticulitis or Incidental Association?

**DOI:** 10.7759/cureus.102794

**Published:** 2026-02-01

**Authors:** Maria J Araujo, Victor A Sato, Precil D Neves, Erico S Oliveira, Leonardo V Pereira, Sara Mohrbacher, Alessandra M Bales, Luciana L Nardotto, Marcella M Frediani, Andrea Santos Galvão, Wares F Medeiros, Américo L Cuvello-Neto, Pedro R Chocair

**Affiliations:** 1 Internal Medicine, Hospital Alemão Oswaldo Cruz, São Paulo, BRA; 2 Internal Medicine, Oswaldo Cruz German Hospital, São Paulo, BRA; 3 Internal Medicine and Nephrology, Hospital Alemão Oswaldo Cruz, São Paulo, BRA

**Keywords:** abdominal infection, case report, covid-19, diverticulitis, persistent fever

## Abstract

This report describes the case of a previously healthy 54-year-old woman who presented with acute sigmoid diverticulitis. Outpatient treatment with ciprofloxacin and metronidazole was initiated. After four days of starting on antibiotics, she returned to the hospital with medication-resistant fever and worsening abdominal discomfort, prompting admission. Intravenous therapy with appropriate antibiotics (ceftriaxone and metronidazole) was initiated, but the fever recurred, suggesting possible treatment failure. On the fourth day of hospitalization, with the onset of mild respiratory symptoms (mild rhinorrhea and nasal voice), a respiratory viral panel was performed, which tested positive for SARS-CoV-2. The patient showed progressive clinical improvement and was discharged after completion of antibiotic therapy. This case highlights the importance of considering viral infections, including COVID-19, in the differential diagnosis of persistent fever during the treatment of diverticulitis.

## Introduction

Acute colonic diverticulitis is a common gastrointestinal condition, accounting for a significant proportion of emergency department visits related to abdominal pain and fever, particularly in middle-aged and older adults [1,2]. The disease results from micro- or macro-perforation of a colonic diverticulum, leading to localized inflammation and, in some cases, abscess formation or peritonitis. Uncomplicated diverticulitis is usually managed conservatively with antibiotics and supportive care, while complicated cases may require invasive interventions [1].

Persistent fever during treatment typically raises concern for complications such as abscess, perforation, or antimicrobial failure. However, during the COVID-19 pandemic, SARS-CoV-2 infection has been increasingly recognized as a cause of gastrointestinal symptoms, including abdominal pain, diarrhea, and fever, sometimes occurring in the absence of prominent respiratory manifestations [3-6].

Several reports have demonstrated that COVID-19 may mimic acute abdominal conditions or alter their clinical presentation, posing diagnostic challenges for clinicians [7-12]. In this context, distinguishing persistent inflammatory fever from treatment failure versus concomitant viral infection becomes clinically relevant. We report a case of acute sigmoid diverticulitis in which persistent fever during appropriate antimicrobial therapy was ultimately explained by concurrent SARS-CoV-2 infection, highlighting an important diagnostic pitfall.

## Case presentation

A 54-year-old woman, previously healthy and a living kidney donor approximately 20 years earlier, presented to the emergency department with a three-day history of unconfirmed fever and left lower quadrant abdominal pain. She denied respiratory symptoms at presentation. Physical examination revealed a patient in good general condition, afebrile, with blood pressure of 118/68 mmHg, respiratory rate of 18 breaths per minute, heart rate of 75 beats per minute, and an axillary temperature of 36.5°C. Abdominal examination showed tenderness on deep palpation of the left iliac fossa, with mild rebound tenderness.

**Figure 1:**
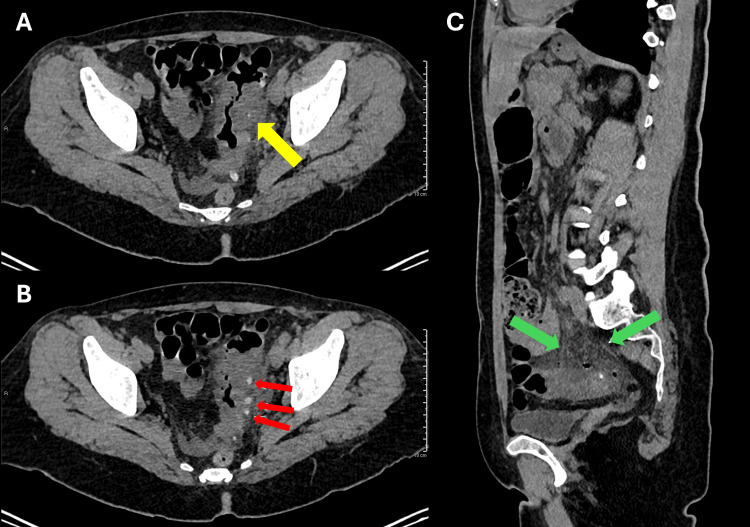
Non-contrast abdominal CT findings consistent with acute sigmoid diverticulitis. (A, B) Axial images showing parietal thickening of a sigmoid diverticulum (yellow and red arrows) and adjacent wall. (C) Sagittal image demonstrating densification of the adjacent mesenteric fat (green arrows).

Laboratory tests showed hemoglobin of 14.6 g/dL, hematocrit of 41.3%, leukocyte count of 10,730/mm³, platelet count of 193,000/mm³, serum creatinine of 0.71 mg/dL, no electrolyte abnormalities, amylase of 56 U/L, lipase of 31 U/L, C-reactive protein of 1.8 mg/dL, aspartate aminotransferase (AST) of 16 U/L, alanine aminotransferase (ALT) of 13 U/L, and a normal urinalysis. Laboratory test results are given in Table 1. Abdominal computed tomography (CT) without intravenous contrast revealed findings consistent with uncomplicated sigmoid diverticulitis, including focal parietal thickening of a sigmoid diverticulum and adjacent mesenteric fat stranding (Figure 1) [1,2].

**Table 1:** Laboratory test results during the hospitalization

Laboratory Test	Reference Range	Day 0	Day 4	Day 7	Day 9
Hemoglobin (g/dL)	11.7 – 14.9	14.6	13.9	12.2	13.2
Hematocrit (%)	35.1 – 45.1	41.3	39.7	35.5	38
Leukocytes (/mm^3^)	3,650 – 8,120	10,730	10,820	5,730	5,560
Platelets (/mm^3^)	163,000 – 343,000	193,000	199,000	186,000	193,000
Creatinine (mg/dL)	0.6 – 1.1	0.71	0.72	-	0.63
C-Reactive protein (mg/dL)	<1	1.8	1.61	1.20	0.55

The patient was initially managed on an outpatient basis with oral ciprofloxacin (500 mg twice daily) and metronidazole (400 mg three times daily). After four days of antibiotic therapy, she returned to the emergency department due to persistent fever, chills, and worsening abdominal discomfort, prompting hospital admission for further evaluation.

**Figure 2:**
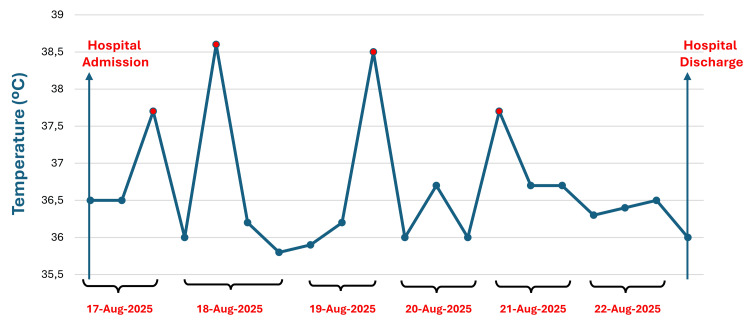
Temperature curve during the hospitalization

On physical examination, blood pressure was 140/70 mmHg, respiratory rate 16 breaths per minute, heart rate 88 beats per minute, and temperature 38.5 °C (Figure 2). Abdominal examination again demonstrated tenderness on deep palpation of the left iliac fossa, without important rebound tenderness. Laboratory evaluation revealed hemoglobin of 13.9 g/dL, hematocrit of 39.7%, leukocyte count of 10,820/mm³, platelet count of 199,000/mm³, serum creatinine of 0.72 mg/dL, no electrolyte disturbances, amylase of 56 U/L, lipase of 31 U/L, C-reactive protein of 1.2 mg/dL, AST of 16 U/L, and ALT of 13 U/L (Table 1). Antibiotic therapy was escalated to intravenous ceftriaxone (1 g twice daily), while metronidazole was continued.

Over the following days, despite improvement in abdominal pain, the patient continued to experience daily episodes of fever. On the fourth day of hospitalization, a contrast-enhanced abdominal CT scan was performed to exclude potential complications of diverticulitis, which showed no evidence of abscess formation, perforation, or disease progression. In addition to fever, the patient reported new mild upper respiratory symptoms, including rhinorrhea and nasal speech.

Given these findings, a respiratory viral panel using reverse-transcription polymerase chain reaction (RT-PCR) was performed, and tested positive for SARS-CoV-2. The patient remained hemodynamically stable, required no respiratory support, and had no recurrence of abdominal pain. She completed a seven-day course of antibiotic therapy, did not receive any specific antiviral treatment, and became afebrile on the same day the COVID-19 diagnosis was established. She was discharged two days later in good clinical condition. At follow-up, she reported complete resolution of abdominal symptoms and no COVID-19-related complications.

## Discussion

This case aims to highlight the diagnostic challenges associated with persistent fever in diverticulitis. Although antibiotic treatment failure or intra-abdominal complications are the main initial concerns, concurrent viral infections should also be considered. SARS-CoV-2 has been shown to cause gastrointestinal symptoms, which may manifest with fever and abdominal pain in the absence of significant respiratory symptoms [3-6]. Abdominal imaging may be normal in some patients with COVID-19; however, a systematic review indicates that more than half of cases exhibit abdominal findings [7].

Persistent fever in diverticulitis during the COVID-19 pandemic should also prompt consideration of non-abdominal causes, including viral infections [8-12]. In patients with diverticulitis undergoing antibiotic treatment, persistent fever or the emergence of new febrile episodes during the clinical course should prompt evaluation with contrast-enhanced abdominal computed tomography to investigate disease-related complications, such as abscess formation or perforation. Other potential sources of fever, including pulmonary or urinary complications that may develop during the course of the illness, should also be considered [13,14].

Besides the clinical overlap, several biological mechanisms support this association: SARS-CoV-2 demonstrates enterotropism, thus disrupting the gut microbiota and inducing microvascular injury, which may contribute to diverticular inflammation. These mechanisms provide a plausible explanation for persistent fever despite appropriate antimicrobial therapy, highlighting the need to consider viral etiologies when the clinical course deviates from expected [8-12]. A previous report has indicated that the clinical appearance of diverticulitis can be altered by a concomitant viral infection and that gastrointestinal symptoms and extra-abdominal complications are common in patients with COVID-19 [12].

Gastrointestinal symptoms, including abdominal pain, nausea, vomiting, and diarrhea, have been reported in approximately 20% of patients diagnosed with COVID-19 [3,4,12]. There are reports of COVID-19 mimicking appendicitis, cholecystitis, and colitis; however, its overlap with diverticulitis is rarely described [9-12]. This rarity highlights the importance of this case report. Previous studies also highlight that persistent fever in diverticulitis should not be immediately interpreted as treatment failure, but rather that extra-abdominal causes, such as viral infections and variations in clinical presentation related to the pandemic, should be considered immediately [8-12].

In patients with diverticulitis under antibiotic treatment, persistent or recurrent fever should prompt contrast-enhanced abdominal CT scanning to rule out complications such as abscess or perforation. Other potential clinical sources of fever, including pulmonary or urinary infections, should also be considered [13,14].

## Conclusions

Persistent fever in patients with diverticulitis is not necessarily a sign of treatment failure or disease progression. In the current epidemiological context, particularly when mild respiratory symptoms are present, COVID-19 infection should be considered as a potential confounder, while maintaining standard evaluation for diverticulitis-related complications. Physicians need to be aware of this diagnostic challenge, as identifying viral coinfection can avoid unnecessary antibiotic use or invasive interventions, ultimately contributing to patient safety and outcomes.
